# ABC transporters of the liver fluke *Fasciola hepatica*

**DOI:** 10.1186/2050-6511-13-S1-A76

**Published:** 2012-09-17

**Authors:** Oliver Kudlacek, Yaprak Dönmez, Thomas Stockner

**Affiliations:** 1 Insitute of Pharmacology, Center of Physiology and Pharmacology, Medical University of Vienna, 1090 Vienna, Austria

## Background

The liver fluke *Fasciola hepatica* is one of the most important parasites affecting animal health all over the world, causing the so called liver fluke disease (fascioliasis). Infections of mammals can only occur via larvae of the fluke, which live on plants in moistly grassland. Therefore infections of humans are rather rare, but infections of animal stock still lead to large financial losses. The drugs of choice used in the treatment of infected animals are benzimidazoles like triclabendazole, which prevent microtubule formation. During the last decades more and more flukes resistant to these drugs have been found. Beside mutations in the target proteins (β-tubulin), detoxificationmechanisms via ABC (ATP-binding cassette) transporters are thought to be responsible for these resistances. Up to date little is known about proteins of the fluke. We therefore try to isolate yet unknown proteins, focusing mainly on ABC transporters, of the fluke, to investigate their potency as putative new drug targets.

## Methods

Adult flukes were collected from bovine liver at a slaughter house in lower Austria. An ABC transporter of *Fasciola hepatica* was cloned from isolated fluke RNA. Heterologous expression was used for further characterization of the transporter in various cell lines. Polyclonal antibodies were raised to allow localization of the transporter by immunfluorescence microscopy.

## Results and conclusions

We could clone a full length ABC transporter consisting of twelve transmembrane domains, showing high homology to members of the B-type family of ABC transporters. Heterologous expression of this *Fasciola hepatica* ABC transporter showed mainly intracellular localization in various cell lines, including mammalian and insect cell lines. Some members of the B-family of ABC transporters, like the mitochondrial transporters, ABCB10, are known to be expressed intracellularly. We have not determined a substrate of the transporter yet, but if the expression pattern holds true for the subcellular expression of the native fluke transporter, this transporter can not be responsible for resistances to anthelmintic drugs in the liver fluke. We have two other ABC transporters identified by sequence comparison and cloned from fluke RNA which will be analyzed in similar ways.

